# Effects of Muse Cell on a Mouse Model With Acute Encephalopathy

**DOI:** 10.1002/brb3.70242

**Published:** 2025-01-19

**Authors:** Tatsuya Kawaguchi, Tetsuji Mori, Kaori Adachi, Jun Fujii, Yoshihiro Maegaki, Fumiko Obata

**Affiliations:** ^1^ Division of Child Neurology, Department of Brain and Neurosciences, Faculty of Medicine Tottori University Yonago Japan; ^2^ Department of Biological Regulation, School of Health Science, Faculty of Medicine Tottori University Yonago Japan; ^3^ Department of Pediatrics Nara Medical University Hospital Kashihara Japan; ^4^ Research Initiative Center, Organization for Research Initiative and Promotion Tottori University Yonago Japan; ^5^ Division of Bacteriology, Department of Microbiology and Immunology, Faculty of Medicine Tottori University Yonago Japan

**Keywords:** acute encephalopathy, cytokine storm, Muse cell, NOD/SCID mouse

## Abstract

**Introduction:**

Acute encephalopathy (AE) in childhood due to a viral infection causes convulsions and altered consciousness, leading to severe sequelae and death. Among the four types of AE, cytokine storm–induced AE is the most severe and causes serious damage to the brain. Moreover, a fundamental treatment for AE has not been established yet. Recently, it has been shown that the administration of multilineage‐differentiating stress‐enduring (Muse) cells, a population of mesenchymal stem cells, improves symptoms in various types of brain injuries when administered in the subacute phase (1–7 days after brain damage). We aimed to examine the effects of Muse cells in a cytokine storm–induced AE animal model using immunocompromised nonobese diabetic/severe combined immunodeficiency (NOD/SCID) neonatal mice.

**Methods:**

We established a modified protocol to induce AE‐like symptoms in NOD/SCID. Then, Muse cells were injected at an acute phase (2–4 h after hyperthermia treatment).

**Results:**

Injection of Muse cells significantly improved body weight gain 1 day after treatment and the survival ratio for 3 weeks.

**Conclusion:**

These effects could be a result of the direct and/or indirect upregulation of IL‐10, an anti‐inflammatory cytokine, in the Muse cell–treated brain. Although non‐Muse cells, a residual cell population in the bone marrow after isolating Muse cells, also improved some symptoms, their effects were weaker than those of Muse cells. Our results indicate that the injection of Muse cells in the acute phase has an effect on AE, suggesting that they exert their therapeutic effects not only in the subacute phase but also in the acute phase.

## Graphical abstract

We developed an acute encephalopathy model with NOD/SCID mice (NOD/SCID‐AE) to evaluate the effects of pluripotent Muse cells in treating AE. Muse cells were effective in weight gain and expression of the anti‐inflammatory cytokine IL‐10 in the very early phase of the disease.

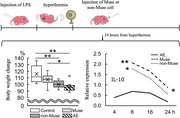

## 1 Introduction

Acute encephalopathy (AE) is reported in children in East Asia due to various viral infections, such as influenza virus, human herpesvirus 6, rotavirus, and coronavirus (Hoshino et al. 2012). AE causes high fever, convulsions, altered consciousness, and often severe sequelae, such as intellectual disability, motor paralysis, intractable epileptic disease, and death. Currently, the detailed mechanisms underlying AE remain unclear. AE is classified into four categories based on the clinical and neuropathological findings: metabolic error–induced AE, cytokine storm–induced AE, excitotoxicity‐induced AE, and AE of unknown etiology (Mizuguchi et al. 2007). Cytokine storm–induced AE is characterized by an increased expression of proinflammatory cytokines, with the most severe symptoms and worst prognosis among all types of AE (Ichiyama et al. 2003). Cytokine storm–induced AE includes acute necrotizing encephalopathy (ANE) (Mizuguchi et al. 2021) as well as hemorrhagic shock and encephalopathy syndrome (HSES) (Rinka et al. 2008). ANE and HSES are characterized by blood–brain barrier (BBB) disruption, leading to cerebral vasogenic edema and neuronal necrosis (Mizuguchi et al. 2007, 2002; Nakai et al. 2003; Kuki et al. 2015). In addition, both ANE and HSES lead to the failure of multiple organs associated with disseminated intravascular coagulation, including the liver and kidneys (Mizuguchi et al. 2007). Currently, there are no radical treatments for AE. Symptomatic treatments, such as injections of adrenocortical steroids, anticonvulsants, and free radical scavengers, exist; however, their effects are limited (Mizuguchi et al. 2021).

Mesenchymal stromal cells (MSCs) are highly heterogeneous. A population of MSCs includes stem cells with a strong capacity for replication in vitro and the ability to differentiate into multilineage cells, such as mesodermal, ectodermal, and endodermal cells (Kuroda et al. 2010; Dominici et al. 2006; Renesme et al. 2022). Therefore, MSC transplantation has attracted attention for the repair of damaged organs for clinical purposes. Within MSCs, there are 1%–3% of pluripotent stem cells called multilineage‐differentiating stress‐enduring (Muse) cells, which can be isolated from human bone marrow–derived MSCs (BM‐MSC) (Kuroda et al. 2010; Wakao et al. 2011). Muse cells selectively accumulate at the site of injury to replace, replenish, and repair injured cells by spontaneous differentiation more efficiently than crude MSCs (Yamada et al. 2018). Moreover, Muse cells have immunoregulatory functions through the secretion of anti‐inflammatory cytokines, such as TGF‐β, IL‐10, and complement C3 (Gimeno et al. 2017; Yabuki et al. 2018).

Muse cells have been used in neuropathological disease models such as lacunar infarction, amyotrophic lateral sclerosis (ALS) and enterohemorrhagic *Escherichia coli*‐associated encephalopathy, or in clinical trials such as ischemic stroke, spinal cord injuries, ALS, and cerebral palsy, all of which show a healing potential of Muse cells in the central nervous system (CNS) (Yamauchi et al. 2015; Shimamura et al. 2017; Park, Borlongan, and Dezawa 2021; Ozuru et al. 2019). These reports prompted us to test Muse cells in the AE mouse model for their beneficial effect on the brain.

Recently, we developed a cytokine storm–induced AE model using ICR mice (ICR‐AE), an immunocompetent mouse line (Kurata et al. 2019). Injection of lipopolysaccharide (LPS) followed by hyperthermia (HT) treatment (AE‐inducing treatment) in Postnatal Day 8 (P8) mice resulted in high mortality, angiogenic edema due to BBB disruption, and activation of astrocytes and microglia in the acute phase (6 h after AE‐inducing treatment). The neuropathological findings of this model were similar to those of ANE and HSES, including necrosis of neurons and astrocytes and focal ischemia. Thus, in the present study, we examined the effects of Muse cells at the acute phase on survival rates and neuropathological brain injuries in an AE mouse model.

## 2 Materials and Methods

### 2.1 Animals

Severely immunocompromised nonobese diabetic/severe combined immunodeficiency (NOD/SCID) mice were purchased from Charles River Laboratories Japan (Yokohama, Japan), housed in a HEPA‐filtered ventilation environment, and maintained under a 12‐h light/dark cycle. The mice were mated, and the day when the pups were delivered was defined as P0. P8 and P9 pups of both sexes were used in this study. All the experiments were conducted in accordance with the Guidelines for Animal Experimentation, Faculty of Medicine, Tottori University, under the International Guiding Principles for Biomedical Research Involving Animals.

### 2.2 Treatments to Induce AE‐Like Symptoms

AE model mice were prepared according to a slightly modified version of a previous study (Kurata et al. 2019; Supporting Information S1). Behavioral seizures in the pups were limited to generalized tonic–clonic seizures, and any other movements that could be recognized as part of a partial seizure were excluded.

### 2.3 Preparation and Injection of Muse and Non‐Muse Cells

**FIGURE 1:**
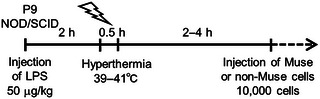
Schematic representation of treatments. The protocol of AE‐inducing treatments was slightly modified for NOD/SCID mouse. For details, see Section 2.

Muse and non‐Muse cells were kindly provided by Dr. Mari Dezawa (Tohoku University, Japan). These cells were prepared from adult human BM‐MSCs (Lonza, MD, USA). Purchased cells were detached and made into cell suspension. Using anti‐SSEA‐3‐conjugated fluorescence‐activated cell sorting, Muse cells were isolated as described previously (Kuroda et al. 2013). Muse cells are stage‐specific embryonic antigen (SSEA)‐3^+^ cells, whereas non‐Muse cells are SSEA‐3^−^ cells (Kuroda et al. 2013). Non‐Muse cells were used as controls. Cells were injected into the retro‐orbital sinus at a dose of 10,000 cells/50 µL with a 31‐gage needle 2–4 h after HT treatment (Yardeni et al. 2011; Figure 1 and Supporting Information S2). Mice injected with Muse and non‐Muse cells after the AE‐inducing treatment were defined as the Muse and non‐Muse groups, respectively. Two groups were used for control groups: age‐matched pups injected with 50 µL of vehicle 2 h after the AE‐inducing treatment (the AE group) and the no treatment group (the control group).

### 2.4 Analysis of BBB Disruption

BBB disruption was analyzed as described previously (Kaya and Ahishali 2011; Morrey et al. 2008; Supporting Information S3). The degree of BBB disruption was determined by dividing the fluorescence intensity by the weight of the brain.

### 2.5 Histological Quantification of the Microglial Morphology

Rabbit anti‐Iba1 antibody (1:1000; Wako Chemicals, Tokyo, Japan) was used for immunohistochemistry. Incubation with secondary antibodies conjugated to biotin (1:200; Vector, Newark, CA, USA), followed by an ABC elite kit (Vector) and Ni/DAB, was conducted according to the manufacturer's instructions.

To analyze microglial morphology, three sections from each mouse were randomly selected. Three animals in each experimental group were analyzed. Tiled images of the entire cortex were obtained using a 40× objective lens equipped with an all‐in‐one microscope (BZ‐X800; Keyence, Osaka, Japan). Using the ImageJ software (https://imagej.net/), grid lines making 100 × 100 µm^2^ were superimposed on images, and three squares were randomly selected (in the dorsal, middle, and ventral cortex using coronal sections or in the anterior, middle, and posterior cortex using sagittal sections), and all cells in the selected squares were analyzed. The ramification index was defined as the ratio of the cell area (Ac, the area outlined by darker blue, the polygon tool in ImageJ) and the projection area (Ap, the polygonal area outlined by teal blue, convex hull analysis in ImageJ) in Figure 4E (Wittekindt et al. 2022).

### 2.6 Analysis of Inflammatory Cytokine Profiles

Pups were perfused with PBS as described in Supporting Information S4. Total RNA was isolated from the brain of each mouse using the RNeasy Plus Universal Mini Kit (Qiagen, Valencia, CA, USA). Inflammatory cytokines (IL‐6, IL‐10, and TNF‐α) mRNAs were measured by quantitative reverse transcriptase–polymerase chain reaction (qRT‐PCR) using the TaqMan probes (Thermo Fisher Scientific). The comparative CT method was used to measure the relative amounts of those cytokine mRNAs normalized with β‐actin mRNA. The expression of each cytokine in each experimental group was compared with the control group.

### 2.7 PCR Screening of Muse/Non‐Muse Cells in the Brain Parenchyma

A human‐specific PV92 Alu sequence localized on chromosome 16 was targeted. Genomic DNA was extracted from the brains after the pus was perfused with PBS, according to the protocol of the GeneElute Mammalian Genomic DNA Miniprep Kit (Sigma Aldrich, St. Louis, MO, USA). PCR was performed as described previously with a slight modification (Comas et al. 2001; Supporting Information S5). The primer sets were expected to produce 129, 443, or 789 bp amplicons. Human genomic DNA (Clontech, Mountain View, CA, USA) was used as a positive control for the PCR reaction. Amplicons were electrophoresed on a 1.5% agarose gel.

### 2.8 Statistical Analysis

For multigroup comparisons, we performed the one‐way ANOVA followed by Tukey's post hoc test (parametric analysis) or the Kruskal–Wallis test followed by the Steel–Dwass test (nonparametric test), depending on the results of the Shapiro–Wilk normality test and Bartlett's test for homogeneity variances to compare the differences among the experimental groups. Survival rates for each group were determined using the Kaplan–Meier method, followed by the Bonferroni test. Statistical significance was set at *p *< 0.05. All statistical analyses were performed using the SPSS Statistics 29.0.1 software (IBM, Tokyo, Japan) or EZR (ver.1.56), a free statistical software package based on R (Kanda 2013).

## 3 Results

### 3.1 Optimization of the AE‐Induction Protocol on the NOD/SCID Mouse

In the present study, a NOD/SCID mouse was used as the host animal to prevent immune rejection of xenografted human cells. Immune responses against LPS and susceptibility to HT treatment differ among mouse strains (van Gassen et al. 2008; Piirsalu et al. 2022). Thus, the experimental conditions established for ICR mice were modified to induce AE‐like symptoms in NOD/SCID mice.

**FIGURE 2:**
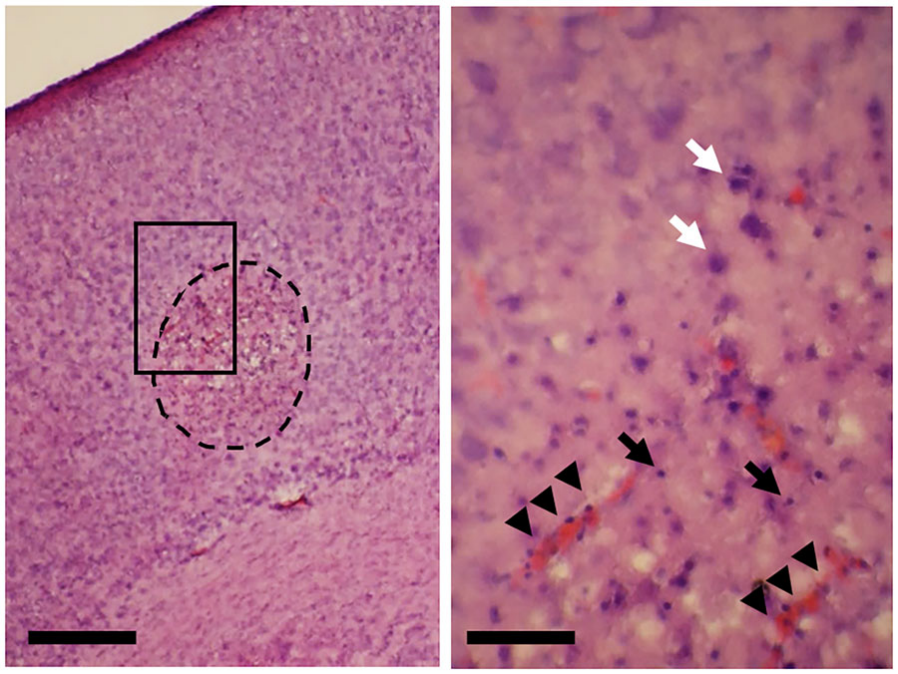
Evaluation of AE‐like histopathology in NOD/SCID mouse. An image of an HE‐staining section from the AE group 24 h after HT treatment is shown. In the cerebral cortex, representative of a region (circled by dots) containing pycnotic neurons (white arrows) is shown. Mononuclear cells (black arrows) are accumulated, and edema is obvious. A high‐magnification image of the boxed area is shown in the right panel. Black arrowheads indicate blood vessels. Scale bar = 500 and 50 µm for the left‐ and right‐hand panels, respectively.

First, the original protocol was applied to P8 NOD/SCID mice. LPS (100 mg/kg) was injected, and HT treatment was followed 2 h after (Figure 1); however, all pups died within 6 h post HT. As 58.8% of P8 ICR pups survived with the same condition, it was more severe in the NOD/SCID (Kurata et al. 2019). When LPS was reduced to 50 mg/kg with other conditions staying the same, the mortality rate was as high as 75% in NOD/SCID. In principle, the NOD/SCID pups were smaller than the ICR pups during the early postnatal stages. The body weights of NOD/SCID at P9 were comparable to ICR pups at P8 (data not shown). Therefore, 50 mg/kg LPS was injected into P9 NOD/SCID mice, resulting in 48% survival post 6 h HT, which was comparable to the ICR‐AE. Moreover, the brain regions contained pyknotic neurons and mononuclear cells (Figure 2, white and black arrows, respectively), as previously reported for ICR‐AE (Kurata et al. 2019). Therefore, P9 NOD/SCID treated with 50 mg/kg of LPS was determined as an AE‐inducing treatment.

### 3.2 Effects of Muse Cells on BBB Disruption and Microglial Activation

BBB disruption is the major lesion in AE. Therefore, the effect of Muse cells on BBB disruption was analyzed 24 h after AE‐inducing treatment. The control and AE groups were used as BBB disruption‐negative and ‐positive controls, respectively.

**FIGURE 3:**
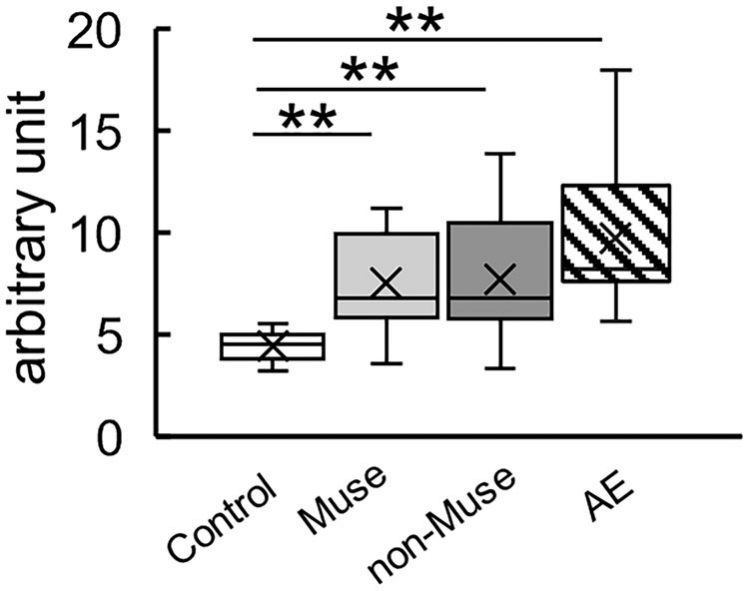
Evaluation of BBB disruption 8 h after AE‐inducing treatment. Fluorescence intensity per 1 g brain was determined (*n* = 8/group). Fluorescence intensity in the Muse, non‐Muse, and AE groups is significantly higher than in the control group, and injection of Muse cells and non‐Muse cells did not significantly improve the BBB disruption. The Kruskal–Wallis test followed by the Steel–Dwass multiple comparison test was performed. **p < 0.01.

The median fluorescence intensities per 1 g of the brain were 4.51, 6.78, 6.80, and 8.25 for the control, Muse, non‐Muse, and AE groups, respectively. The fluorescence intensity was significantly higher in the Muse, non‐Muse, and AE groups than in the control group, but there was no significant difference among the Muse, non‐Muse, and AE groups (Figure 3).

**FIGURE 4:**
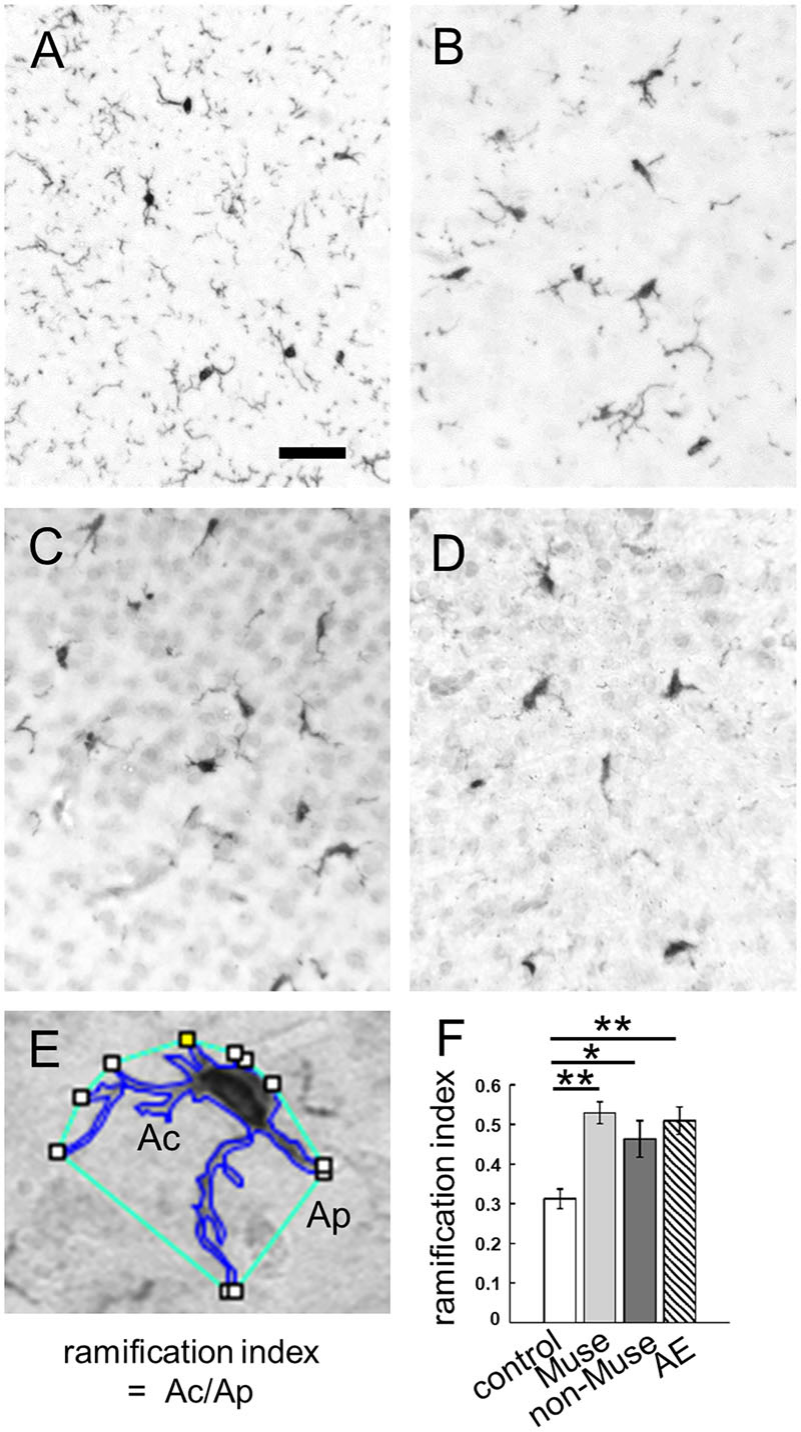
Activation of microglia. Images of immunohistochemistry with an anti‐Iba1 antibody are shown. The sections from the control (A), the AE (B), the Muse (C), and the non‐Muse groups (D) 24 h after the AE‐inducing treatment. Cell bodies of Iba1‐immunopositive cells in the AE group, Muse group, and non‐Muse group are slightly larger than in the control group. (E) A definition of ramification index. The area of Iba1‐positive cell (Ac) was divided by the projection area (Ap). (F) The value of the ramification index was significantly higher in the Muse, non‐Muse, and AE groups than in the control group. Three mice in each group (average 26.41 ± 6.46 cells/animal) were analyzed. Error bars indicate standard deviation. One‐way ANOVA followed by Tukey's post hoc test was performed. *p < 0.05. Scale bar = 50 µm for A–D. **p < 0.01.

The activation of glial cells is another alteration observed in AE (Kurata et al. 2019). The activation of Iba1‐immunopositive microglia in the NOD/SCID mice was much lower than that in the ICR mice. In addition, the microglia had slightly larger cell bodies in the AE group than in the control group at 24 h post‐AE‐inducing treatment (Figure 4A,B; Kurata et al. 2019). Notably, the microglia in the Muse and non‐Muse groups were slightly activated (Figure 4C,D). Thus, the ramification index was calculated: the Iba1 immunopositive cellular area (Ac) is divided by the foot process projection area (Ap) (Figure 4E; Wittekindt et al. 2022). Resting microglia have ramified processes, but activated microglia have a hypertrophic morphology so highly branched (ramified) resting microglia present small values, whereas activated microglia exhibit larger values. The Muse, non‐Muse, and AE groups exhibited significantly larger values than the control group, but there was no significant difference among the Muse, non‐Muse, and AE groups (Figure 4F).

### 3.3 Effect of Muse Cells on Body Weight Gain and Survival

**FIGURE 5:**
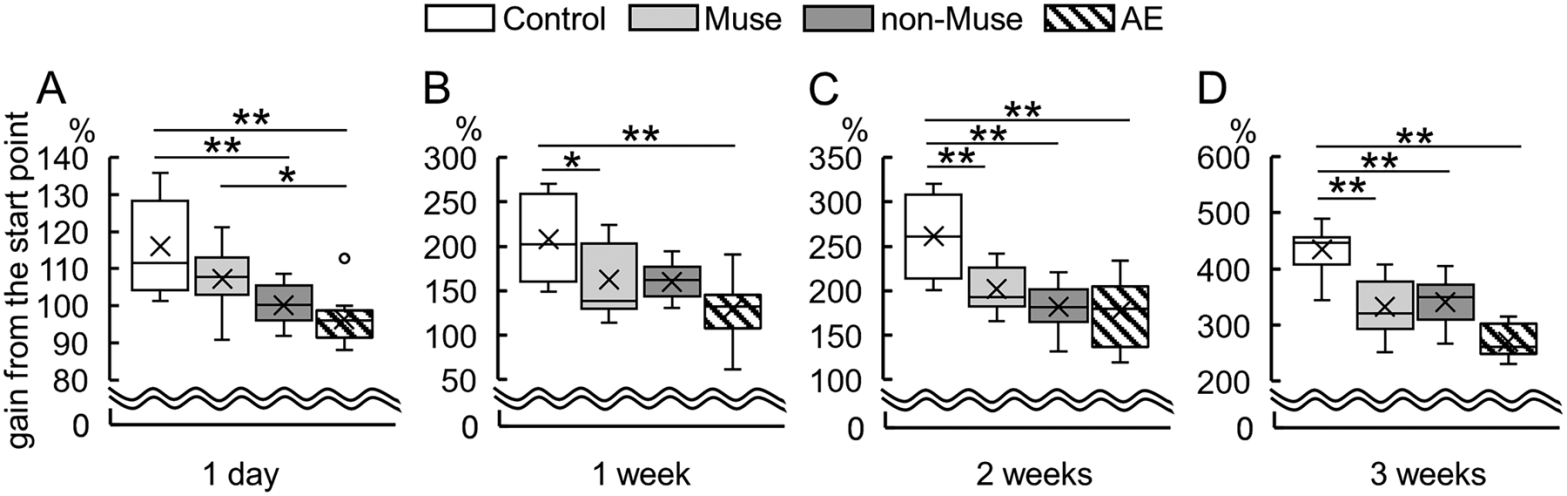
Effect of Muse cells on body weight gain. Body weight gain was evaluated for 3 weeks post‐AE‐inducing treatment. In each experimental group (*n* = 16), the body weight at the beginning of the AE‐inducing treatment is set as a benchmark. (A) One day after treatment, the body weight gain of the non‐Muse and the AE groups was significantly less than that of the control group. Moreover, there was no significant difference between the control and the Muse groups, and the AE group had significantly less body weight gain than the Muse group. (B) One week after the AE‐inducing treatment, the body weight gain of the Muse and the AE groups was significantly less than that of the control group. Two (C) and three weeks (D) after the AE‐inducing treatment, the body weight gain of all the groups with AE‐inducing treatment, regardless of human cell injection, was significantly less than that of the control group. Kruskal–Wallis test followed by Steel–Dwass multiple comparison test was performed. *p < 0.05; **p < 0.01.

Next, the long‐term effects of Muse cells on body weight gain and the survival ratio were analyzed. First, the body weight in each experimental group was measured at 1 day, 1, 2, and 3 weeks post‐AE‐inducing treatment. The body weight gain was normalized to pre‐LPS weight. One day post‐AE‐inducing treatment, the body weight gain of both the non‐Muse and AE groups was significantly lower than that of the control group. In contrast, the Muse group showed significantly higher body weight than the AE, and it was at the equivalent level to the control (i.e., no significant difference between Muse and control) (Figure 5A).

One week after AE‐inducing treatment, the body weight gain in both the Muse and AE groups was significantly lower than that in the control group (Figure 5B). The body weight gain in all groups was significantly lower than that in the control group in 2 and 3 weeks (Figure 5C,D). Throughout the survival period, body weight gain was not significantly different between the Muse and non‐Muse groups.

**FIGURE 6:**
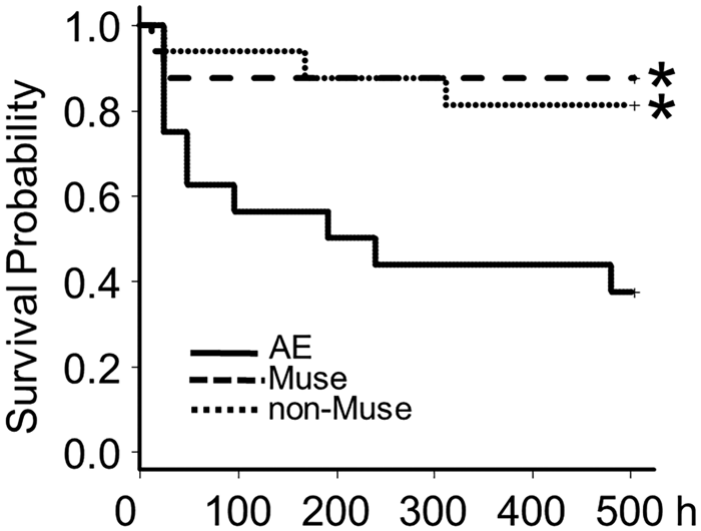
Effect of Muse cells on the survival rate. Administration of Muse or non‐Muse cells improved the survival ratio after the AE‐inducing treatment over 3 weeks (*n* = 16). There were significant differences between the Muse and the AE groups and between the non‐Muse and the AE groups. Kaplan–Meier analysis followed by Bonferroni test was performed. *p < 0.05.

The survival ratios were determined for 3 weeks (504 h) after the AE‐inducing treatment. The survival was checked every 2 h for 8 h after the AE‐inducing treatment and once a day thereafter (Figure 6). The survival ratios were 87.5%, 81.3%, and 37.5% in the Muse, non‐Muse, and AE groups, respectively, at 504 h. The survival ratios in the Muse and non‐Muse groups were significantly higher than in the AE group. However, there were no significant differences between the Muse and non‐Muse groups (Figure 6).

### 3.4 Effect of Muse Cells on mRNA Expression of Inflammatory Cytokines in the Brain

**FIGURE 7:**
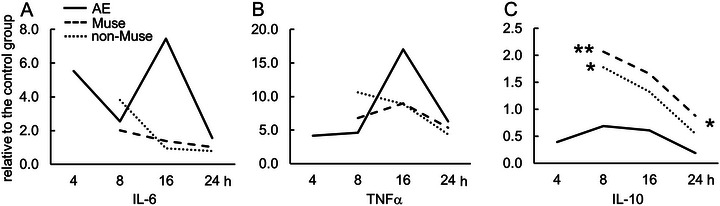
Effects of Muse cells on inducing mRNA expression of inflammatory cytokines. Expression of inflammatory cytokine mRNAs over 24 h after the AE‐inducing treatment was determined by qRT‐PCR. In each experimental group (*n* = 8), mRNA expression levels of proinflammatory cytokines, IL‐6 (A) and TNF‐α (B), and anti‐inflammatory cytokine, IL‐10 (C), were normalized with that of the control group. Expression profiles of proinflammatory cytokines were not significantly different among experimental conditions. Expression of IL‐10 was significantly upregulated in the Muse group and the non‐Muse group at 8 h, and it was significantly higher in the Muse group at 24 h after the AE‐inducing treatment. Kruskal–Wallis test followed by Steel–Dwass multiple comparison test was performed. *p < 0.05; **p < 0.01.

As shown above, NOD‐SCID‐AE presented BBB disruption and microglial activation, which are the major characteristics of cytokine storm–induced AE. Thus, mRNA induction of proinflammatory (IL‐6 and TNF‐α) and anti‐inflammatory (IL‐10) cytokines was evaluated by qRT‐PCR at 4, 8, 16, and 24 h after the AE‐inducing treatment (Figure 7). The expression levels of these cytokines were normalized to those in the control group. Cytokine expression levels at 4 h after the AE‐inducing treatment were excluded because the effects of Muse/non‐Muse cell injection were expected to be detected at relatively later time points (Gimeno et al. 2017).

At 16 h after the AE‐inducing treatment, expression of both IL‐6 and TNF‐α mRNA reached a peak in the AE group, and it declined subsequently. The average mRNA expression levels of these proinflammatory cytokines were lower in the Muse and non‐Muse groups than in the AE group at 16 h after the AE‐inducing treatment. However, there were no significant differences between the experimental groups (Figure 7A,B). In contrast, the expression level of IL‐10 mRNA in the Muse and non‐Muse groups was significantly higher than that in the AE group at 8 h (Figure 7C). Later, the expression of IL‐10 declined but was significantly higher in the Muse group than in the other experimental groups (Figure 7C).

### 3.5 Existence of Muse/Non‐Muse Cells in the Brain

As described above, the use of Muse cells improved several aspects of AE symptoms. We hypothesized that Muse cells infiltrate the brain parenchyma and reduce cytokine storm by directly and/or indirectly upregulating IL‐10. To examine this hypothesis, we attempted to detect human cells with histological and PCR‐based techniques.

An anti‐STEM121 monoclonal antibody has been widely used to detect cytoplasmic proteins in human cells engrafted in mice (Kamei et al. 2023). We performed screening with the anti‐STEM121 antibody on the sections from the Muse and non‐Muse groups 24 h and 2 months after AE‐inducing treatment, but no STEM121‐immunopositive cells were detected (Supporting Information S4, data not shown).

**FIGURE 8:**
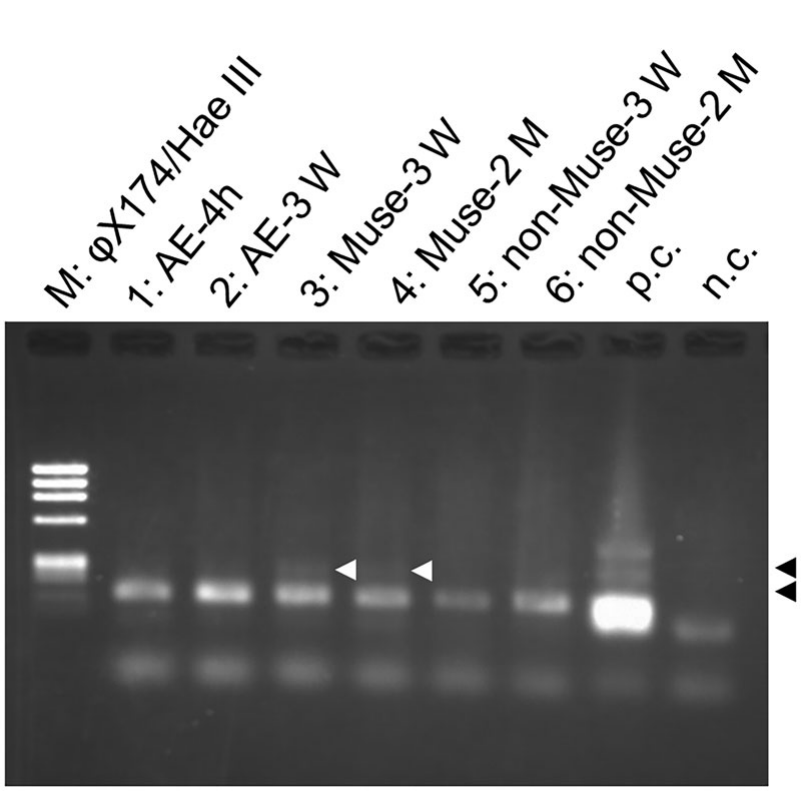
PCR screening for administrated human cells at 3 weeks (3 W) and 2 months (2 M) after the AE‐inducing treatment. Very faint bands of Alu sequence were detected in some mice those administrated with Muse cells (white arrowheads). For positive control (p.c.), human genomic DNA was used as a template DNA, and two bands were detected (black arrowheads). For negative control (n.c.), no DNA was added in a reaction tube. Lower two bands were expected as primer dimers. (M) φX174/Hae III digest, (1) the AE group 4 h after the AE‐inducing treatment, (2) the AE group 3 W, (3) the Muse group 3 W, (4) the Muse group 2 M, (5) the non‐Muse group 3 W, and (6) the non‐Muse group 2 M.

Therefore, a more sensitive and higher‐throughput method was employed: a PCR‐based screening targeting human‐specific Alu sequence. No amplicons of the Alu sequence were detected at 24 h after AE‐inducing treatment (data not shown). In contrast, very faint amplicons were detected in the Muse group but not in the non‐Muse group at 3 weeks and 2 months after AE‐inducing treatment (Figure 8).

## 4 Discussion

In this study, we applied an AE‐inducing protocol to NOD/SCID mice and examined the therapeutic effects of administered Muse cells against AE during the acute phase.

We confirmed whether the AE‐inducing protocol could be applied to immunodeficient mouse strains. NOD/SCID mice lacked functional T and B cells with no detectable immunoglobulins in the sera (Shultz et al. 1995). The NOD/SCID mice show lower NK cell activity, lack complement activity, and have macrophages with less maturation/activity (Takenaka et al. 2007). Therefore, they are excellent recipients of human cells. Although they are immunocompromised, they retain their inflammatory capacity. The NOD/SCID mice produce IL‐6 upon inoculating the *Coxiella burnetii* antigen complex (Sukocheva et al. 2010). An enterohemorrhagic *E. coli* infection model using NOD/SCID mice showed signs of inflammation with weight reduction, astrocyte activation in the brain, and death. However, 1 of 10 of the *E. coli* dose used in immunocompetent mice was sufficient to induce inflammation (Ozuru et al. 2019; Amran, Fujii, Kolling, et al. 2013; Amran, Fujii, Suzuki, et al. 2013).

Morphological activation of microglia, astrocytes, and astrocytic clasmatodendrosis are critical pathological changes in AE and are seen in the ICR‐AE model (Kurata et al. 2019). In contrast, the NOD/SCID‐AE model exhibited activation of microglia, judging from the ramification index (Figure 4E,F) in AE, Muse, and non‐Muse groups but no obvious signs of activation for astrocytes (i.e., no astrocytic clasmatodendrosis) are observed. Thus, in the immunocompromised NOD/SCID mice, the AE model seems to present a partial morphological glial activation. This might be similar to reduced glial cell responses, which occur after stroke in pharmacologically and genetically immunosuppressed mice (Weber et al. 2022). However, transient upregulation of proinflammatory cytokines was evident in NOD/SCID mice after the AE‐inducing treatment, which might increase the chance of LPS entering the brain parenchyma caused by BBB disruption (Kurata et al. 2019). The microglia are the primary source of proinflammatory cytokines. The astrocytes are also reactive against LPS and produce TNFα (Landoni et al. 2010). Moreover, LPS administration in mice induces microglial activation, which, in turn, activates astrocytes via cytokine production (Liddelow et al. 2017). As described above, the NOD/SCID mice have limited function of macrophage/monocytic lineage cells, including microglia. Thus, in our NOD/SCID‐AE model, the activation of microglia and astrocytes may have been limited. Nevertheless, the upregulation of proinflammatory cytokines and the infiltration of mononuclear cells into the brain parenchyma, where pyknotic neurons reside, were evident in the NOD/SCID‐AE model (Figure 2). These results indicated that the AE‐inducing protocol developed by Kurata et al. (2019) is universal for a variety of animals.

Body weight gain significantly improved in the Muse group 1 day after AE‐inducing treatment (Figure 5A). This may represent a prolonged IL‐10 effect at 24 h in the Muse group, which was not observed in non‐Muse group (Figure 7C). Nevertheless, both the Muse and non‐Muse groups showed similar trends in weight gain and survival rate, which may be a result of the transient upregulation of IL‐10 in the acute phase. IL‐10 exerts anti‐inflammatory effects by inhibiting the production of proinflammatory cytokines in LPS‐stimulated macrophages. As LPS itself does not induce IL‐10, some mediators must be involved in the upregulation of IL‐10 in both the Muse and non‐Muse groups. Rat bone marrow–derived Muse (rBM‐Muse) cells induce expression of IL‐10 in TNFα‐treated intestinal epithelial cells (Sun et al. 2020). Furthermore, rBM‐Muse cells produce higher levels of IL‐10 in an inflammatory microenvironment than rBM‐MSCs do, which include non‐Muse cells and a fraction of Muse cells (Sun et al. 2020). Thus, the human BM‐Muse cells used in the present study may be directly and/or indirectly involved in the high expression of IL‐10 in the AE‐induced brain. As a direct effect, cells that migrate into the CNS parenchyma may exert effect by secreting substances or interacting with other parenchymal cells, whereas as an indirect effect, a secreted product from Muse or Muse‐influenced blood cells to produce cytokines or other substances may enter the CNS and alter parenchymal cells. Both Muse and non‐Muse groups transiently exhibited increased IL‐10 expression in the brain 8 h after the AE‐inducing treatment. However, higher IL‐10 expression was maintained for 24 h only in the Muse group. Thus, the maintenance of high expression levels of IL‐10 should have led to improved body weight gain in the Muse group 1 day post‐AE‐inducing treatment.

The survival ratio was significantly improved in the Muse and non‐Muse groups. Muse cells express both SSEA‐3 (a human pluripotency marker) and CD105 (a marker for bone marrow stroma cells, MSCs) on their surface, and Muse cells used in the present study were isolated from bone marrow targeting SSEA‐3 expression. MSCs isolated from the bone marrow, in addition to Muse cells, have therapeutic effects against several types of injury (Li et al. 2021). MSCs are a highly heterogeneous population (Mabuchi et al. 2021), and the non‐Muse cells used in the present study included pluripotent SSEA‐1^+^ and SSEA‐4^+^ cells (Shirazi et al. 2017; Lee et al. 2021). Thus, non‐Muse cells still contain pluripotent cell populations, which may improve the survival ratio.

We think there are two factors, namely dose and timing, to consider for a successful Muse administration. It has been shown that Muse cells can be a promising treatment for many diseases and injuries in which a single organ or limited area is damaged, such as cerebral infarction, spinal cord injury, and acute myocardial infarction (Abe et al. 2020; Kajitani et al. 2021; Takahashi et al. 2023; Yamada et al. 2018; Noda, Nishigaki, and Minatoguchi 2020). Sphingosine 1‐phosphate (S1P) secreted by damaged cells is recognized by Muse cells to infiltrate damaged organs (Yamada et al. 2018). On the other hand, multiple organs, such as the brain, liver, and kidneys, are involved in patients with AE. Under AE conditions, the administered Muse cells may have been recruited to multiple organs by the secreted S1P, and the number of Muse cells recruited to the brain must have been small. Thus, the administration of a larger number of Muse cells may have improved outcomes in our NOD/SCID‐AE model in the acute phase.

The administration of Muse cells in the current study may have been too early for cells to infiltrate the damaged brain. Indeed, very faint amplicons of the Alu sequence were detected in the brain at 3 weeks and 2 months, but not at 24 h after the AE‐inducing treatment. In this study, Muse cells were administered for 2–4 h after HT treatment, which is considered the acute phase. Previous reports have shown that Muse cells administered in the subacute phase for 1–7 days after brain lesions successfully mitigated the condition (Yamada et al. 2018; Yamauchi et al. 2015; Uchida et al. 2016; Suzuki et al. 2021). Furthermore, in a mouse model of *E. coli*‐associated encephalopathy, it was shown that the administration of Muse cells at 48 h rather than 24 h postinfection increased infiltration of Muse cells into the damaged brain (Ozuru et al. 2019). Moreover, S1P upregulation occurs relatively late after brain damage. The concentration of S1P in the brain after ischemia changes with time; S1P concentration transiently decreases on Day 3 after infarction, then increases and remains at a high level for 14–21 days (Kimura et al. 2008). Thus, the S1P concentration in the damaged brain may not be sufficient for the recruitment of transplanted Muse cells during the acute phase. Therefore, the dual administration of Muse cells in the acute and subacute phases may be effective in managing AE.

## 5 Conclusion

We established an AE mouse model using NOD–SCID mice, which allowed human Muse cell transplantation. Transplantation of Muse cells in the acute phase of AE significantly improved the survival ratio and body weight gain, probably via transient upregulation of IL‐10. This can improve the management plan for AE.

## Author Contributions

**Tatsuya Kawaguchi**: data curation, investigation, validation, writing–original draft, visualization, software, funding acquisition, formal analysis. **Tetsuji Mori**: writing–review and editing, investigation, methodology, validation, conceptualization. **Kaori Adachi**: investigation, validation. **Jun Fujii**: methodology, conceptualization. **Yoshihiro Maegaki**: writing–review and editing, conceptualization, methodology, supervision, resources, project administration. **Fumiko Obata**: writing–review and editing, conceptualization, methodology, supervision.

## Ethics Statement

All animal experiments were conducted in accordance with the Guidelines for Animal Experimentation, Faculty of Medicine, Tottori University, under the International Guiding Principles for Biomedical Research Involving Animals (23‐Y‐10). We attest that the contents of the current manuscript and study align with the Wiley ethics guidelines.

## Consent

The authors have nothing to report.

## Conflicts of Interest

The authors declare no conflicts of interest.

### Peer Review

The peer review history for this article is available at https://publons.com/publon/10.1002/brb3.70242

## Supporting information



Supporting Information

## Data Availability

Data is available upon request to corresponding authors.
