# Supplementary material for: Effects of Muse Cell on a Mouse Model With Acute Encephalopathy
Source: Brain Behav. 2025 Jan 19;15(1):e70242. doi: 10.1002/brb3.70242 (PMC11743977; doi:10.1002/brb3.70242)
Supplement: Supplementary file 1 — Supporting Information [file BRB3-15-e70242-s001.docx]

Supplementary material 1

LPS (*E. coli* serotype O127:B8; Sigma Aldrich, St Louis, MO, USA) was dissolved in sterile pyrogen-free phosphate buffered saline PBS (pH 7.0) at a concentration of 5 mg/mL. LPS was intraperitoneally injected at a dose of 50 or 100 mg/kg, 2 h before HT treatment (Fig. 1). The pups were placed in a transparent container and heated using a heat lamp (Eun et al., 2015)(Kurata et al., 2019). The rectal temperature of the pups was continuously monitored using a rectal probe (RET-4; Physitemp Instruments, Clifton, NJ, USA) connected to a multipurpose thermometer (BAT-10; Physitemp Instruments). The rectal temperature was increased by 0.5°C every 2 min and was maintained at >39°C for 30 min. Heating was discontinued when a convulsion was noted or when the rectal temperature exceeded 41.0°C. HT treatment was resumed when the rectal temperature decreased to <40°C and seizure interruption was confirmed. After 30 min of HT treatment, the pups were placed in a cool container containing water-soaked paper towels until their rectal temperatures returned to normal (33-35°C) and then placed back in their cages.

Supplementary material 2

The prepared cells were stored at 100,000 cells/tube and frozen at -80°C until use. Immediately before injection, cells were thawed and adjusted to 100,000 cells/0.5 mL of a vehicle: Dulbecco's Modified Eagle Medium (Thermo Fisher Scientific, Waltham, MA, USA) with 0.1 mg/mL kanamycin sulfate (Thermo Fisher Scientific), 1 ng/mL FGF-2 (Miltenyi Biotec, Bergisch Gladbach, Germany), and 10% Fetal Bovine Serum (FBS, HyClone, South Logan, UT, USA). P8 or P9 pups were anesthetized by hypothermia on ice.

Supplementary material 3

To analyze BBB disruption in the AE model pups, sodium fluorescein (SF; Wako, Tokyo, Japan) was dissolved in PBS at a concentration of 100 mg/mL, and SF was intraperitoneally injected at a dose of 1 mg/g 45 min before HT treatment. To wash out the SF from the vessels, the pups were perfused with PBS at a volume of 2 mL/g, as described below. The brains were removed and cut along the sagittal plate. The left cerebral hemisphere was weighed, homogenized in 1 mL of sterile PBS, and mixed with 1 mL of 60% trichloroacetic acid by vortexing for 2 min to remove proteins. Homogenized samples were kept at 4°C for 30 min and centrifuged with 18,000 x g at 4°C for 10 min to obtain the supernatant (Kaya & Ahishali, 2011) (Morrey et al., 2008). The fluorescence intensity of 250 μL of the supernatant was measured using a microplate reader (excitation, 440 nm; emission, 525 nm; ARVO X3, PerkinElmer, Waltham, MA, USA).

Supplementary material 4

For histological analysis, pups were anesthetized by hypothermia on ice, and mice older than 3 weeks were anesthetized by intraperitoneally injecting a mixture of medetomidine (0.3 mg/kg), midazolam (4 mg/kg), and butorphanol (5 mg/kg) in PBS. Then, they were transcardially perfused with PBS, followed by 4% paraformaldehyde in PBS (4% PFA). The brains were removed and post-fixed with 4% PFA overnight.

For hematoxylin-eosin (HE) staining, fixed brains were cryoprotected with 20% sucrose/PBS overnight. The brains were embedded in Super Cryo Mount (Muto Pure Chemicals, Tokyo, Japan) and snap-frozen on dry ice. Then, 30-µm-thick coronal sections were cut using a cryostat, and they were attached to the gelatin-coated glass slides.

For immunohistochemistry, fixed brains were embedded in paraffin, cut into 6-µm-thick sections, and attached to silane-coated glass slides. For immunohistochemical human cell detection, anti-STEM121 monoclonal antibody (Y40410, Takara Bio Inc., Kusatsu, Japan) was used.

Supplementary material 5

Human genome Alu-specific PCR reaction was performed as follows. The first round PCR reaction (PV-92A primer: 5’-AACTGGGAAAATTTGAAGAGAAAGT-3,’ and PV-92B primer 5’-TGAGTTCTCAACTCCTGTGTGTTAG-3,’ amplified by 45 cycles) was followed by the second round PCR reaction with a nested primer set (PV92A-2^nd^: 5’-AGATACATTTCAGTAAGGTT-3,’ and PV92B-2^nd^: 5’-TGTTTAGAGAGGGAATTCT-3,’ 1 μL of the first round amplicon was amplified by 20 cycles).
